# Primary prevention of ovarian cancer by salpingectomy: that's one small step for a surgeon, one giant leap for patients

**DOI:** 10.1007/s00432-023-04697-z

**Published:** 2023-03-29

**Authors:** Ingo B. Runnebaum, Angela Kather

**Affiliations:** Department of Gynecology and Reproductive Medicine, Jena University Hospital, Friedrich Schiller-University Jena, Am Klinikum 1, 07747 Jena, Germany

Ovarian cancer (OC) is a difficult-to-detect tumor and as such mostly diagnosed in advanced stages, resulting in poor prognosis. OC accounts for one-quarter of female reproductive organ malignancies, but for half of deaths caused by these cancers. Guideline-compliant treatment comprises complete cytoreductive surgery including multivisceral resections, followed by combination chemotherapy and maintenance therapy with a PARP inhibitor and/or an angiogenesis inhibitor. This multimodal treatment is strenuous for patients and entails high costs for the health care system.

Since the randomized controlled screening trial UKCTOCS including 202 562 women receiving ultrasound screening and CA125 measurements (Menon et al. 2021) did not proof reduction of ovarian cancer deaths, early detection of OC will not be available in the near future. Consequently, options for primary prevention of OC are of fundamental value.

Lifetime ovulatory years show a strong positive association with risk for OC (Fu et al. 2023). Consequently, high parity and long-lasting breastfeeding or oral contraceptive use can be perceived as primary prevention. Though, such influences may collide with aspects of female health and way of life, beyond OC prevention.

Pathologists needed a long time to identify the precursor lesions for the most prevalent and aggressive subtype of OC, high-grade serous ovarian cancer (HGSOC). Finally, it turned out that HGSOC does not originate from the ovaries, but from the fallopian tubes (Kurman and Shih 2016), particularly from their fimbriae (Fig. 1). The scientific offset was the identification of serous tubal intraepithelial carcinomas (STICs) in prophylactically removed fallopian tubes of women with hereditary breast and ovarian carcinoma (carriers of BRCA germline mutation), but also in fallopian tubes of women with sporadic ovarian cancer. Whole-exome and tumor evolutionary analysis revealed that the majority of tumor-specific alterations in HGSOC, mostly affecting BRCA, TP53, and PTEN, are already present in STICs and confirmed STICs as the precursor lesions (Labidi-Galy et al. 2017). Based on this new perception, the WHO issued the classification “tubo-ovarian” cancer. Additionally, development of STIC and high-grade serous cancer (HGSC) following inactivation of BRCA, TP53, and PTEN was demonstrated in mouse models. Early salpingectomy in these mice could prevent development of HGSC. Population-based cohort studies in Sweden found a significantly reduced OC risk for women of the general population, who had a history of salpingectomy on benign indication (Falconer et al. 2015). Recently, a study from Canada analyzed the effect of comprehensive implementation of opportunistic salpingectomy on OC incidence (Hanley et al. 2022). This investigation showed that the incidence of HGSC can be reduced effectively.

**Fig. 1:**
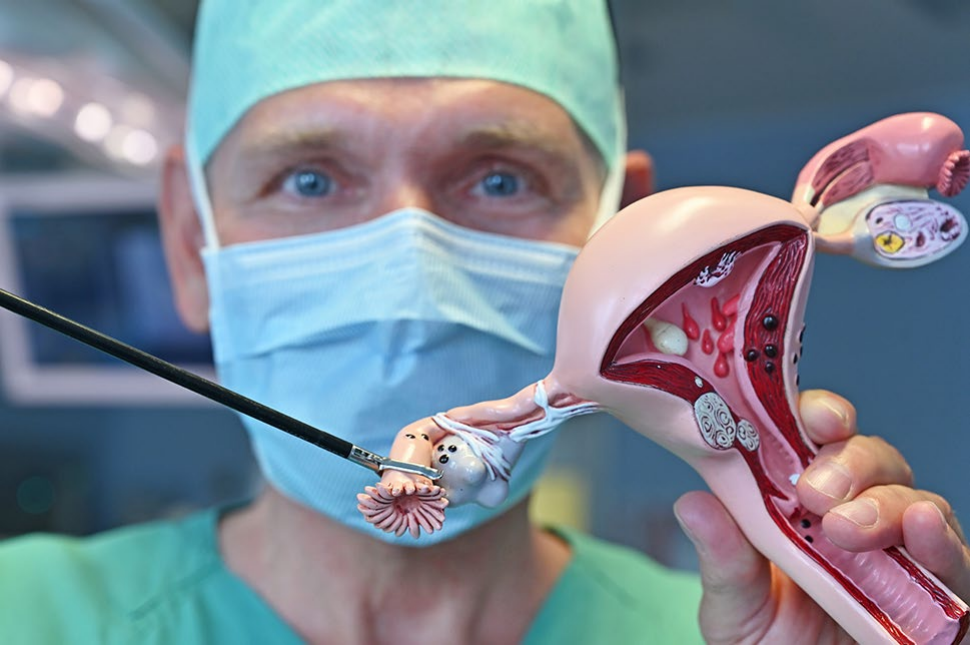
Fallopian tube with fimbriae held by a laparoscopic instrument (surgeon: Ingo B. Runnebaum), model by GPI Anatomicals, Lake Bluff, IL, USA (with kind permission by GPI)

As a consequence of this conclusive scientific picture, some national gynecologic societies published recommendations for opportunistic salpingectomy at the time of benign pelvic surgery in appropriate patients. In laparoscopic or open surgery, to perform an OS is a small step, a simple procedure of less than 5 min. However, many gynecologic societies still hesitate to universally recommend opportunistic salpingectomy (Ntoumanoglou-Schuiki et al. 2018). This is formally justified by the lack of prospective, randomized studies on the protective effect of salpingectomy regarding OC risk. However, such studies are hardly feasible, due to the rather low incidence of OC and the long time period between benign surgery and typical onset age of OC. Further concerns relate to a potentially increased risk for peri-operative complications and for impairment of ovarian function. However, several retrospective investigations proved the safety of opportunistic salpingectomy when performed by experienced surgeons respecting the blood supply of the ovaries. Prospective studies on such issues are currently ongoing (Gelderblom et al. 2022). From the economic perspective, additional costs and surgery time needed for salpingectomy at hysterectomy are neglectable compared to costs and effort of OC treatment. This has been shown in models pertaining to the American healthcare system (Naumann et al. 2021) and would probably also hold true in other countries.

Regardless of the indecisiveness of national professional societies, gynecological surgeons and their patients have increasingly adopted the concept of opportunistic salpingectomy during the past 10 years, as currently demonstrated for Germany in this journal (Runnebaum et al. 2023). The majority of the interviewed gynecologists are convinced about the benefit of opportunistic salpingectomy regarding OC prevention. Benign hysterectomies in premenopausal women are combined with salpingectomy in the majority of cases and tubal ligation is gradually replaced by complete removal of fallopian tubes. German gynecologists have thus accomplished a “de facto standard”, which demands a de jure standard as soon as possible, to provide legal basis for pelvic surgeons, the doctor/patient relationship, and the informed consent.

The time has come to think about implementation of opportunistic salpingectomy at every abdominal surgical intervention with accessible fallopian tubes. This would increase the number of women, who could benefit from OC risk reduction and could lead to a noticeable reduction in OC incidence. Feasibility has recently been shown for elective laparoscopic cholecystectomy in an Austrian study (Tomasch et al. 2020). Appropriate interdisciplinary cooperation should be established and general surgeons should be informed and instructed accordingly.

An international register would be desirable to document the number of opportunistic salpingectomy procedures, side-effects, and HGSC incidence in the cohort with OS, to compensate for unachievable prospective RCTs. Instead of postponing OS for decades until level 1 evidence from RCTs may become available, we may as well act in the best interest of the women at risk and trust for the time being level 2 evidence from large population-based data and promote in a heuristic manner OS as one measure of primary prevention of a lethal disease.

## Data Availability

Data are available upon reasonable request.
